# Valorisation of salmon backbones: Extraction of gelatine and its applicability in biodegradable films

**DOI:** 10.1016/j.heliyon.2024.e34373

**Published:** 2024-07-16

**Authors:** Revilija Mozuraityte, Laura Rodríguez-Turienzo, Raquel Requena, Rasa Slizyte

**Affiliations:** a SINTEF Ocean, Brattørkaia 17C, NO-7010, Trondheim, Norway; b IRIS Technology Solutions, Carretera d’Esplugues 39-41, 08940, Cornellá, Spain

**Keywords:** Gelatin, Salmon, Backbone, Valorisation, Rest raw materials, Biodegradable films

## Abstract

Salmon backbones make up about 10 % of the total fish weight and contain valuable proteins, collagen and lipids that can be used for marine ingredients production. Gelatine is derived from the collagen fraction and this study evaluated how different fractionation and extraction procedures can affect the yield and composition of extracted gelatine. Fractionation by mild thermal treatment of backbones (10 min in 40–42 °C) leads to structural changes of muscle, which improves separation of meat from bones and gives better yield of de-muscled backbone fractionation compared to mechanical meat removal. The highest yield of the gelatine (9.3 ± 0.3g dry gelatine from 100g de-muscled backbone dry material) was obtained from mechanically de-muscled backbones. De-muscled backbones were pre-treated with alkaline (0.04 N NaOH) followed by EDTA and 10 % ethanol for de-calcification and lipid extraction, respectively. Gelatine from pretreated backbones was extracted with 60 °C water. The amount of gelatine amino acids (sum of hydroxyproline, proline and glycine) was 43.4 ± 0.2 % of all amino acids in the gelatine. Extracted backbone gelatines showed film-forming ability. Gelatine films were obtained by casting procedure. Resulted salmon backbone 6 % gelatine and 30 % sorbitol films showed properties (e.g. water vapour permeability, colour difference, transparency value) similar to films obtained with commercial gelatine, indicating the capability of the extracted gelatines for its valorisation as edible coatings or bio-based film layers in packaging.

## 1 Introduction

According to the FAO the amount of rest raw materials generated from the fish industry represents up to 70 percent of the initial weight of the fish [1]. The major fraction of rest raw materials is currently processed into fish meal/oil and used for feed production, but these processes and products have low profitability. Therefore, devising strategies for full utilization of the fish by processing rest raw materials for production of new commodities for human consumption are important both for fishery and processing industry [2]. Salmon backbones make up about 10 % of the total fish weight [3] and are rich both in protein and lipids. Therefore they can be used for high quality oil and protein production [4]. Industrially obtained backbones usually contain a significant amount of muscle (38–55 %) that may be removed using de-boners. The removed muscle may have different applications e.g. fish burger production, but some challenges like metal taste and oxidative instability have been observed [5]. Due to the large amount of collagen present in fish bones it can be used for the extraction of food grade gelatine [6,7].

Recently the demand and use of collagen and gelatine have expanded in foods, pharmaceuticals and cosmetics. Traditionally, the commercially available collagen was produced from porcine and bovine skin and bones. However, due to certain biosafety concerns (bovine spongiform encephalitis, foot and mouth diseases) and religious restrictions (e.g. Kosher standards, Halal certification) associated with these protein sources, as well as the increased focus on utilizing fish rest material, there has been an increasing focus on producing gelatine/collagen from alternative raw materials [2,8], such as fish skin, scales, bones and fins [[9], [10], [11]].

Generally, gelatine is generated by partially hydrolyzed collagen. Conversion of collagen into soluble gelatine can be achieved by a pre-treatment step, followed by controlled hydrolysis by heating the collagen in either acid, alkali or enzymatic hydrolysis [8,12]. Extracted gelatine properties and yields depend on the pre-treatment method [13]. Acid and alkaline pre-treatment is used for swelling of collagen and removal of non-collagenous protein.

Since fish bones are rich in calcium, demineralization prior to gelatine extraction is crucial to improve the yield, purity and gel strength of the gelatine. Fish gelatine from cold water fish (cod, salmon) usually have poor gelling ability and their gelling temperature is usually below 8–10 °C [14]. The main constraint related to fish collagen is the establishment of a cost-efficient extraction process for their commercial exploitation.

Gelatine properties depend on the characteristic of the raw material source (Table 1) and the method of extraction used. Due to higher amount of hydroxyproline, mammalian gelatine has higher melting and gelling points compared to marine gelatine (Table 1). However, thickness for single fish gelatine films from various sources has been reported in the same range as the thickness for single mammalian gelatine films. Lower light transmission for fish gelatine films compared to mammalian (Table 1) was attributed to higher double bonds structure that present in some amino acid compounds of gelatine such as glutamine, tyrosine and phenylalanine [12]. Lower water vapour permeability for single fish gelatine films was attributed to higher hydrophobicity due to lower proline and hydroxyproline contents. Synthetic plastic packaging material is currently being replaced by edible and biodegradable packaging materials to avoid their negative effects on the environment and human health [15]. Bio-based polymeric films obtain from natural resources like gelatine, have been studied as alternatives for synthetic food packaging. Major challenges of biodegradable films, especially protein films, are fragility of the material, high water solubility and reduced water vapour permeability. These drawbacks can be overcome by using plasticizers such as glycerol and sorbitol while making the film [16].

**Table 1:** Properties of fish and mammalian gelatine.

Gelatine source	Melting/gelling temperature^a^	Hydroxyproline content^b^, residues/1000 total amino acid residues	Bloom value^c^, g	Gelatine-based films		
				Thickness^c^, mm	Vissible Light Transmission^c^	Water Vapour permeability^a^ (10^−10^ g/m s Pa)
Fish gelatine	Low	50–79	0–270	0.05–0.12	28.4–90.5	0.006–2.1
Mammalian gelatine	High	∼90	130–308	0.04–0.11	63.9–88.8	0.8–9.7

a - [12].

b - [11].

c - [42].

After industrial filleting salmon backbones contain significant amount of muscle which is not rich in collagen (0.3–0.7 %) [17,18]. Moreover, the yield of gelatine from bones was observed to be much higher compared to the yield from muscles [19]. In addition, salmon backbones contain higher amounts of oil compared to lean fish backbones and their use for production of collagen and gelatine is not widely studied. Therefore, in the present work we developed technology allowing diversified utilization of salmon backbones. Two technologies for removal of muscle proteins from salmon backbone for production of protein rich ingredients and oil were tested, followed by extraction of gelatine from de-muscled bones with five technologies. The applicability of the extracted salmon backbone gelatine for biodegradable film formulation was also studied.

## 2 Material and methods

Fresh backbones after filleting farmed salmon (*Salmo salar* L.) at Salmar AS processing plant (Frøya, Norway) were collected and transported chilled to SINTEF Ocean (Trondheim, Norway) within 8–12 h. At SINTEF Ocean fresh backbones were placed in the freezer (−27 °C) and kept till the experimental day (1–3 months). At experimental day, backbones were thawed at 4 °C over night.

All chemical reagents used in this study were of analytical grade and bought from VWR Norway.

### 2.1 Chemical composition analysis

The moisture content in the samples was determined gravimetrically after drying at 105 °C until constant weight of samples was achieved (typically 24 h) [20]. Ash content was determined after heating dried samples at 590 °C for 12 h [20]. Moisture and ash analyses were performed in triplicates.

### 2.2 Protein amount and amino acid composition

Total nitrogen (N) was determined by CHNS–O elemental combustion system (Costech Instruments ECS 4010) and crude protein was estimated by multiplying total N by a factor of 5.55 (conversion factor for gelatine). The measurements were performed in quadruplicates.

The amino acid profile in freeze-dried ground material was analysed by a HPLC system (Agilent Infinity 1260, Agilent Technologies) coupled to an on-line post-column derivatization module (Pinnacle PCX, Pickering laboratories, Mountain View, CA, USA), using ninhydrin (Trione) as a derivatization reagent and a Na + -ion exchange column (4.6 × 110 mm, 5 μm). 18 Standard amino acids, ammonia and taurine were quantified from standard curves measured with amino acid standards. Prior to the analysis, the samples were hydrolyzed in 6 M HCl containing 0.4 % mercaptoethanol for 24 h at 110 °C (HCl hydrolysis). Glutamine and asparagine were converted to glutamic and aspartic acid, respectively. Cystein was quantified as cystine (Cys-Cys). The samples were filtered via micro filter, the pH was adjusted to 2.2 and the samples were further diluted with a citrate buffer (pH 2.2) for the HPLC analysis. All buffers, reagents, amino acid standards and the column were obtained from Pickering laboratories (Mountain View, CA, USA). HCl and mercaptothion was obtained from Sigma-Aldrich.

### 2.3 Total lipid amount

The extraction and determination of total lipids from the samples were performed according the method described by Bligh and Dyer [21]. The analyses were performed in duplicates.

### 2.4 Fractionation of backbones

Fractionation of the backbones into muscle and de-muscled backbones was performed by two technologies. **"Mechanical treatment"** was based on mechanical scraping of the raw muscle from the backbones, while technology "**Thermal treatment"** included mild heating of vacuum-packed backbone placed for 10 min in 40–42 °C water bath to ease the removal of muscle from backbones. The obtained muscle-free backbone fractions were used for gelatine extractions.

### 2.5 Gelatine extraction

**Fig. 1:**
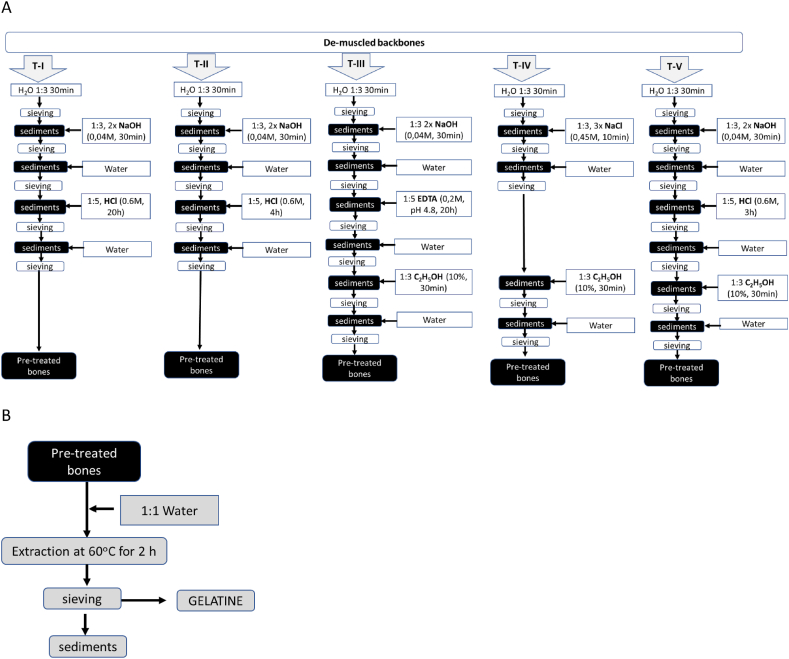
Gelatine extraction technologies (T) used for salmon de-muscled bones. A: Five different extraction technologies (TI -TV) were used with different pretreatments: de-muscled backbones were washed with water (H_2_O) at ratio 1:3 for 30min. After washing backbones were pre-treated by sodium hydroxide (NaOH, 0.04 M), or salt (NaCl, 0.6 M), or hydrochloric acid (HCl, 0.6 M), or ethylenediaminetetraacetic acid (EDTA, 0.2 M pH 4.8). After each pretreatment, drained bones were washed with water. 10 % ethanol solution (C_2_H_5_OH) was used to extract lipids. B - After pretreatment, bones were mixed with warm water (1:1 water 60 °C) for 2 h to extract the gelatine.

Five [5] gelatine extraction technologies from de-muscled backbones obtained after mechanical and thermal treatment were tested (Fig. 1). The applied technologies were based on already published extraction methods, but several modifications were introduced.

The five [5] gelatine extraction technologies contained different pre-treatment steps (Fig. 1A). De-muscled bone pre-treatment in all technological solutions was performed at 4 °C. Technologies I, II and V (T-I, T-II and T-V, respectively) are based on the technology describer by Arnesen and Gildberg [22] and Arnesen and Gildberg [23]. The de-muscled bones were washed with water to remove surface oils from the bones. Washed bones were incubated twice with cold sodium hydroxide solution (0.04 N) for 30 min each to remove other proteins, carbohydrates as well as other unwanted impurities (e.g. lipids) soluble at high pH. The hydroxide solution was drained off. After washing out NaOH with cold water, incubation with HCl acid (0.6 M) was performed for 20 h (T-I), 4 h (T-II) or 3 h (T-V). The acid solution was drained, and the samples were washed with cold water. In technology T-V the incubation with 10 % ethanol solution for 30 min was performed to remove/wash out lipids. The ethanol solution was drained, followed by wash of the bones with cold water.

Wang and Regenstein [24] obtained that EDTA provided the best calcium removal with minimal collagen/gelatine removal. Therefore, in Technology III after treatment with sodium hydroxide (Fig. 1), for calcium removal an incubation of bones with 0.2 M ethylenediaminetetraacetic acid (EDTA) pH 4.8 solution for 20 h was performed. The EDTA solution was drained, followed by bone wash with cold water to remove the rest of EDTA and incubation with 10 % ethanol solution to remove the rest of lipids. The ethanol solution was drained, followed by wash of the bones with cold water. The pre-treated bones were mixed with warm water to extract the gelatine as described above. The technology IV were based on the gelatine extraction method described by Kołodziejska, Skierka [25] where water washed bones were incubated three times with NaCl (0.45 M) solution for 10 min, and after draining of salt, bones were washed with cold water followed by incubation with 10 % ethanol solution for 30 min. The ethanol solution was drained, followed by washing the bones with cold water.

To extract gelatine, all pre-treated bones were mixed with warm water (50–60 °C) and incubated for 2 h in 60 °C water bath with periodical manual stirring approx. each 30 min (Fig. 1B). All tested technological solution were performed with two parallels.

### 2.6 SDS-page electrophoresis

Molecular weight distribution of extracted gelatines was analysed by SDS-PAGE electrophoresis according to the instructions provided by manufacturer (C.B.S. Scientific Company, USA) using ClearPAGE SDS Gel 4–20 % gels and High Molecular Weight Standard (220-53 kDa). The gels were stained using InstantBlue Protein Stain.

### 2.7 Film formation

Gelatine films were obtained by a casting procedure. Backbone gelatine film formulations with 6 % gelatine, and 30 % Sorbitol (Panreac, Spain) were mixed for 45 min at 70 °C and dried at 30 °C in an oven overnight. Commercial gelatine films were obtained from cold water fish skin powder (teleostean gelatine, Sigma Aldrich, Spain) and were also obtained by casting using the same formulation but using Glycerol (Panreac, Spain) instead of Sorbitol as plasticiser. The film formation ability was determined by the capability of the dried formulations to form stand-alone films.

### 2.8 Water vapour permeability (WVP)

WVP of films was determined gravimetrically using the ASTM Method E96/-96 M. Samples (discs of 40 mm diameter) were hermetically sealed (with Teflon seals) in a glass permeation cell containing silica gel. Permeation cells were placed in a desiccator containing distilled water, thus obtaining a relative humidity (RH) gradient equal to 100 % (assuming that the RH on silica gel is negligible). The water vapour transfer through the exposed film area (9.62 cm^2^) was measured from the cell weight gain as a function of time. Cells were weighed using a four-digit balance every 24 h over a 5-day period, after steady state vapour flow had been reached. Triplicate of each formulation were tested and WVP was calculated from the following equation [1]:

(1) $$WVP\;\left(\frac{g}{msPa}\right)=\frac{\text{slope}\;\text{of}\;\text{the}\;\text{weight}\;\text{gain}\;\text{vs}\;\text{time}\;\left(\frac{g}{s}\right)\cdot \;\text{film}\;\text{thickness}\;\left(m\right)}{exposed\;area\;\left(m^{2}\right)\cdot \Delta P\;\left(\text{Pa}\right)}$$

where ΔP is the water vapour pressure differential across the film (2642 Pa at 22 °C). Film thickness was measured by using a digital micrometre at six random locations for the average thickness measurement.

### 2.9 Colour

For colour measurements, a spectrocolorimeter (Datacolor Xpress Spectrocolorimeter) was used. After zero calibration, a reference measurement was done by using a white ceramic plate which served as bottom layer for the gelatine film measurements. Colour of the samples was expressed as L* coordinate (lightness/brightness), a* value (redness/greenness) and b* value (yellowness/blueness). Total difference of colour (ΔE*) was calculated using equation [2] according to Nilsuwan et al. [26]:

(2) $$\Delta E*=\sqrt{{\left(\Delta L*\right)}^{2}{\left(\Delta a*\right)}^{2}{\left(\Delta b*\right)}^{2}}$$

where Δ*L*∗, Δ*a*∗ and Δ*b*∗ are the differences between the colour parameter of the samples and those of the white standard.

### 2.10 Transparency

The transparency of films was measured at 600 nm by using an UV-VIS spectrophotometer (Lambda 35 UV/VIS spectrometer, PerkinElmer). The films were cut into a rectangular piece and placed directly in a UV–Vis spectrophotometer cuvette, using an empty cuvette as the reference. Three replicates were tested for each film and the absorbance values were also converted to transmittance values using the Lambert–Beer equation [27]. The transparency value of film was calculated using the equation [3]:

(3) $$\text{Transparency}\;\text{value}=\frac{-\;\mathrm{Log}\;T600}{x}$$

where T_600_ is the fractional transmission at 600 nm and x is the film thickness (mm). A higher transparency value indicates reduced light transmission across the film or a higher opacity. Thus, the greater transparency value represents the lower transparency of the film.

### 2.11 Statistical analysis

Minitab 18 (Minitab Inc., PA, USA) was used for statistical analysis. Statistical significance of the experimental data was verified by using one-way ANOVA and the Tukey method for comparison of means. The statistical significance level was set to p < 0.05.

## 3 Results and discussion

### 3.1 Fractionation

Salmon bones contain approx. 46 % of dry material (DM) where lipids made up approx. 56 % of DM, proteins 34 % and ash approx. 10 % of DM. Fractionation of the backbones into muscle and de-muscled bones was performed by two fractionation technologies: mechanical and thermal treatment and the yields of fractions are given in Table 2. Thermal treatment, where backbones were heated till 40–42 °C water bath for 10 min, led to more muscle removal from backbones compared to mechanical treatment (Table 2). This indicates that mild thermal treatment leads to structural changes of muscle, which allows better separation of meat from bones and gives better fractionation yield.

**Table 2:** Mass balance of fractions obtained by mechanical and thermal fractionation of backbones.

Fractionation technology	Mass balance, g/100g wet weight		Mass balance, g/100g dry weight	
	Bones	Muscle	Bones	Muscle
Thermal	51 ± 4	49 ± 4	59 ± 4	41 ± 4
Mechanical	57 ± 5	43 ± 4	64 ± 5	36 ± 4

The obtained de-muscle bone fractions, using thermal or mechanical fractionation, had similar composition (Table 3). However, a slightly higher ash content in the thermally de-muscled bone fraction was observed, which is due to better removal of muscle from the bone compared to mechanical fractionation. The muscle fraction obtained could be used as raw material for production fish mince products like fish burgers or sausages, for production of marine lipids, protein hydrolysates [4] or taste neutral protein production [28].

**Table 3:** Chemical composition of de-muscled bones.

Fraction	Amount, g/100g wet weight			
	dry material	lipid	protein	ash
Thermal de-muscled bone	48.8 ± 1.7	26.4 ± 0.8	13.3 ± 0.9	6.4 ± 1.2
Mechanical de-muscled bone	48.1 ± 0.9	26.8 ± 1.2	12.9 ± 0.7	4.8 ± 1.1

### 3.2 Gelatine extraction from de-muscled backbones

Five extraction technologies were used to extract the gelatine from de-muscled bones obtained after thermal and mechanical fractionation and the yields are given in Table 4. The initial muscle separation technology (mechanical vs thermal treatment) did not influence the yield of obtained gelatine significantly for the five different extraction technologies. However, the different extraction technologies resulted in different yields of gelatine. The highest yield of gelatine was obtained using the technological solution T-III (significant at p < 0.05). All the technological solutions gave higher yields of gelatine compared to the 2.5 % gelatine yield from salmon backbones reported by Tamir and Abdullah [29] and Xiong et al. [30].

**Table 4:**
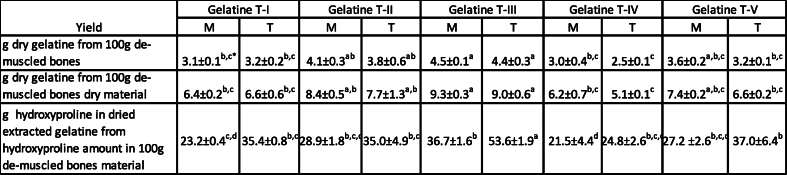
Yields of gelatine extracted from de-muscled salmon bones (mechanically (M) and thermally (T) fractionated) by five different technologies (Fig. 1). The yield is given as values presented as average of measurements (n = 2) ± SD.

⁎ Values followed by the same letter in the same row are not significantly different at p < 0.05.

The difference between T-I and T-II is exposure time in acid solution which was 20 and 4 h, respectively. Five times longer acid treatment gave slightly lower yield of gelatine obtained by T-I compared to T-II (Table 5). Both Zhou and Regenstein [31] an Blanco, Vázquez [32] also observed that prolonged treatment with acid could cause loss of collagen, even using a weak acid with a low H concentration at a low temperature.

**Table 5:**
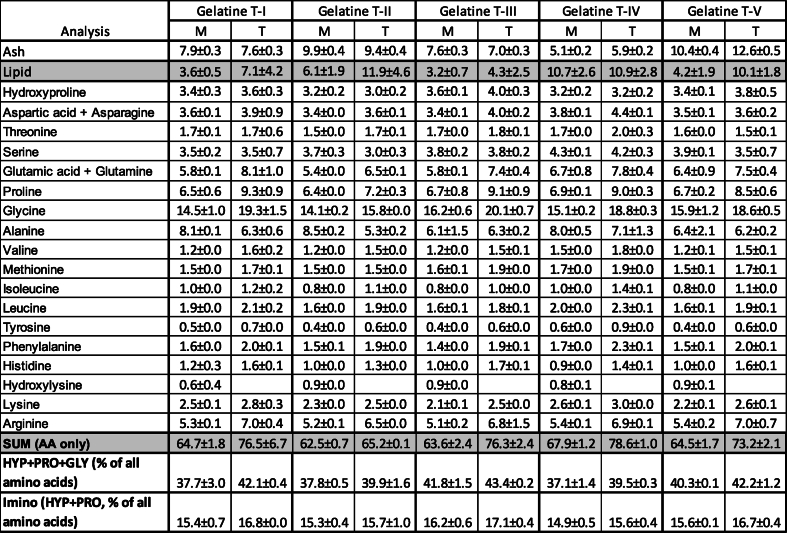
Chemical composition (lipid, ash and amino acid amount) of gelatines extracted by five technologies (T-I, T-II, T-III, T-IV, T-V) from de-muscled bones using mechanical (M) or thermal (T) fractionation. Lipids and amino acids are given g/100g dry gelatine powder. HYP – hydroxyproline, PRO – proline, GLY – glycine.

Chemical composition of obtained gelatines (amount of lipid, ash, and amino acids) is given in Table 5. The obtained gelatines contained significant amount of lipids (3.2–11.9 %). There was a clear trend that gelatines extracted from thermally separated bones contained slightly more lipids compared to the gelatine extracted from mechanically separated bones for all the five extraction technologies. Thermal treatment of backbones could lead to more interaction between remaining lipids and proteins and therefore gelatine contained slightly higher amount of lipids. To reduce lipid amount in the biomass, solvent extraction of lipids with e.g. hexane [33] from raw material could be performed. But use of hexane would increase requirements for safety of processing plant and could limit its applicability in industrial scale. Technology II gave gelatines with lower amount on lipids. However, statistically no significant (p > 0.05) influence of extraction technology on the amount of lipids in the final gelatine was obtained.

Marine lipids are oxidative unstable and the threshold value for oxidation products from marine omega-3 fatty acids are relatively low. Therefore, the gelatines with this amount of marine lipids may have limited applicability and limited shelf-life. Technology T-III, which gave the highest yield of gelatine (Table 2), also resulted in the gelatine with the lowest amount of lipid. Slightly lower lipid amount was obtained in the gelatine extracted using T-V, compared to gelatine obtained by T-II. The difference between those two technologies was an additional ethanol treatment step in technology V and this could lead to slightly lower lipid amount in the gelatine. Longer ethanol treatment time or other alcohol type e.g. butanol, isopropanol [34,35] could lead to better separation of lipids and lower amount of lipids in the final gelatine.

Gelatines extracted using T-II and T-V contained 9.4–9.6 and 10.4–12.6 % of ash, respectively (Table 4). High content of ash was due to salt formation because of the backbone treatment with hydroxide followed by incubation with hydrochloric acid before extraction of the gelatine. The type and amount of salt in the gelatine could affect the functional properties of the gelatine [36]. Higher amount of salt could reduce light transmission of gelatine films and affect colour.

The sum of amino acids abundant in gelatine; sum of hydroxyproline, proline and glycine, was also relatively high in T-III extracted gelatine, but similar as in other treatments. The acid treatment swell due to repulsive forces among collagen molecules, however this treatment may also lead to draining of loosened collagen during the extraction [7]. The amount of imino amino acids (hydroxyproline and proline) (14.9–17.1 %) in the gelatines from salmon de-muscled bone was similar to gelatine extracted from salmon skin (16.6 %) reported by Arnesen and Gildberg [22] and cod skins (15–18 %) [37]. It was observed that gelatine obtained from thermally treated bones contained higher percentage of both HYP + PRO + GLY and imino amino acids compared to gelatine extracted from mechanically separated bones, but this difference was not significant.

**Fig. 2:**
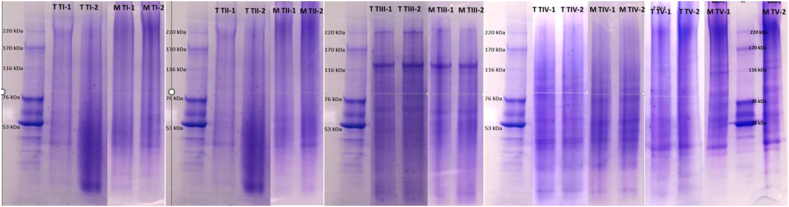
SDS-PAGE electrophoresis pattern of gelatines extracted by different technologies (T I–V) form mechanically (M) and thermally (T) treated backbones.

The SDS -page electrophoresis pattern of extracted gelatines (Fig. 2) indicated that initial pre-treatment (thermal versus mechanical) of backbones did not influence peptides size significantly. However, the extraction technology played a significant role for the size distribution of obtained gelatines. Both gelatines obtained from fish backbone after thermal and mechanical fractionation by using technology T-III contained large peptides in the range from 116 till 220 kDa. Gelatines extracted using technology T-V contained peptides in the range bellow 170 kDa. All other gelatines had peptides with a size lower than 76 kDa. This indicates that gelatines extracted with technology T-III would exhibit better rheology properties, as dynamic storage modulus, amount of helix, melting and gelling temperatures of cold water fish gelatines correlates and increased with increasing molecular weight of gelatine peptides, especially close to 250 KDa [38,39].

Based on the analysed quality parameters (yield, amount of oil in extracted gelatines, molecular weight distribution) it can be stated that extraction technology T-III (M-TIII and T-TIII) was the best among the evaluated technologies. Based on these findings it was decided to use gelatines M-TIII and T-TIII to evaluate film forming properties.

### 3.3 Gelatine-based films characteristics (film forming ability, water vapour permeability (WVP), colour and transparency

Despite the extraction method employed, all backbone gelatines showed film-forming ability. In general, all gelatine films showed dispersion of small particles and a whitish colour, probably due to the presence of some insoluble residues and lipids resulting from the gelatine extraction process. Gelatines with the highest yield and lowest lipid content, were obtained by extraction technology T-III, and were therefore selected for their physical characterization and comparison with commercial gelatine films (Table 6).

**Table 6:** Physical properties of the different gelatine coatings developed from gelatine samples: M -T III – gelatine from salmon backbone extracted using T III extraction technology (see Fig. 1) from mechanically fractionated backbones and T-T III – gelatine from salmon backbone extracted using T III extraction technology (see Fig. 1) from thermally fractionated backbones. Where WVP - water vapour permeability, ΔE - colour difference, T – Transparency value.

Film formulation		WVP·10^−^^1^^0^ (g/m.s.Pa)	ΔE	Transparency value (T)
Backbone gelatine	M T-III	0.9 ± 0.2a	4.2 ± 0.2b	6.28 ± 0.12c
	T T-III	1.1 ± 0.3a	5.0 ± 0.3c	2.79 ± 0.20b
Commercial gelatine		1.1 ± 0.1a	3.1 ± 0.1a	0.16 ± 0.06a

Regarding the WVP of gelatine-based films no significant differences were observed between fish backbone gelatine extracted using T-III from thermal fractionated backbones-compared to commercial warm gelatine films (Table 5), thus it seems that the impurities observed do not affect the water barrier properties of the films.

WVP of backbone gelatine films were comparable to those described for other similar films like, gelatine-starch films (1.08 ± 0.01 g/cm^2^/day), as reported by Jagannath et al. [40]; surimi based edible films (1.69 ± 0.06 10^−^^10^ gm^−1^s^−1^Pa^−1^) as described by Shiku et al. [41], and slightly higher than those reported by Gautam et al. [16] for fish meat films (0.3862 ± 0.0049 g/cm^2^/day).

**Fig. 3:**
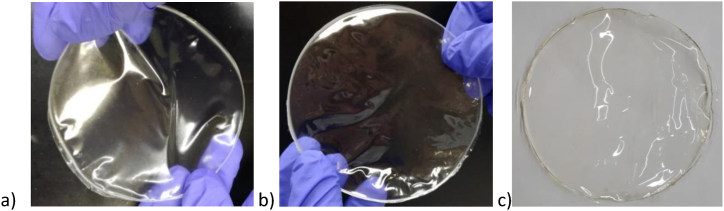
Examples of stand-alone films obtained with Backbone gelatine a) M T-III and b) T T-III and c) films obtained with commercial gelatines.

Moreover, T T-III films (fish backbone gelatine extracted using T-III from thermal fractionated backbones) showed higher colour differences in comparison with commercial gelatine (Table 5). Based on a visual assessment and the transparency values, T T-III films were more transparent than M T-III (fish backbone gelatine extracted using T-III from mechanically fractionated backbones) ones, probably due to a lower amount of insoluble particles when the backbone was thermally separated. Examples of stand-alone films obtained with Backbone gelatine given in Fig. 3 where a) M T-III and b) T T-III and c) films obtained with commercial gelatines.

## 4 Conclusions

This study presents a technological concept to transfer backbones obtained from salmon filleting industry into several high-quality ingredients and products. Fractionation of the backbones into muscle and de-muscled bones using mild heating of the backbones (up to 40 °C) facilitated the easier removal of muscle and could be an alternative the mechanical removal of the muscle from salmon backbones. Muscle fraction may be processed into high quality marine oil and protein rich fractions. It was proved that both mechanical and thermal pre-treatment of backbones lead to muscle-free bones fraction, which can be used for gelatine extraction.

More than 50 % of the hydroxyproline present in de-muscled salmon bones can be extracted by choosing the right pre-treatment and gelatine extraction technology. By using a solution of 10 % ethanol the amount of lipids can be reduced in extracted gelatine, but there is still a significant amount of lipids in the produced gelatine. To ensure proper shelf-life of the gelatine product the lipids need to be stabilised or removed by additional technological step. The amino acid composition indicated that extracted gelatine from salmon bones contained similar amount of hydroxyproline and proline as gelatines from salmon skins.

Irrespective the extraction method employed, all extracted backbone gelatines showed film-forming ability. Films formulated with the extracted gelatines with higher yield and lower lipid content (M T-III and T T-III) resulted in films with similar properties to those obtained with commercial gelatine, indicating the capability of the extracted gelatines for its valorisation as edible coatings or bio-based film layers in packaging.

## CRediT authorship contribution statement

**Revilija Mozuraityte:** Writing – original draft, Investigation, Formal analysis. **Laura Rodríguez-Turienzo:** Writing – original draft, Methodology, Formal analysis, Data curation. **Raquel Requena:** Writing – review & editing, Formal analysis, Data curation. **Rasa Slizyte:** Writing – original draft, Validation, Methodology, Formal analysis, Data curation.

## Declaration of competing interest

The authors declare that they have no known competing financial interests or personal relationships that could have appeared to influence the work reported in this paper.
