# Supplementary figures and images for: Valorisation of salmon backbones: Extraction of gelatine and its applicability in biodegradable films
Source: Heliyon. 2024 Jul 16;10(14):e34373. doi: 10.1016/j.heliyon.2024.e34373 (PMC11324808; doi:10.1016/j.heliyon.2024.e34373)

**SDS page gels for gelatines extracted form salmon backbones**


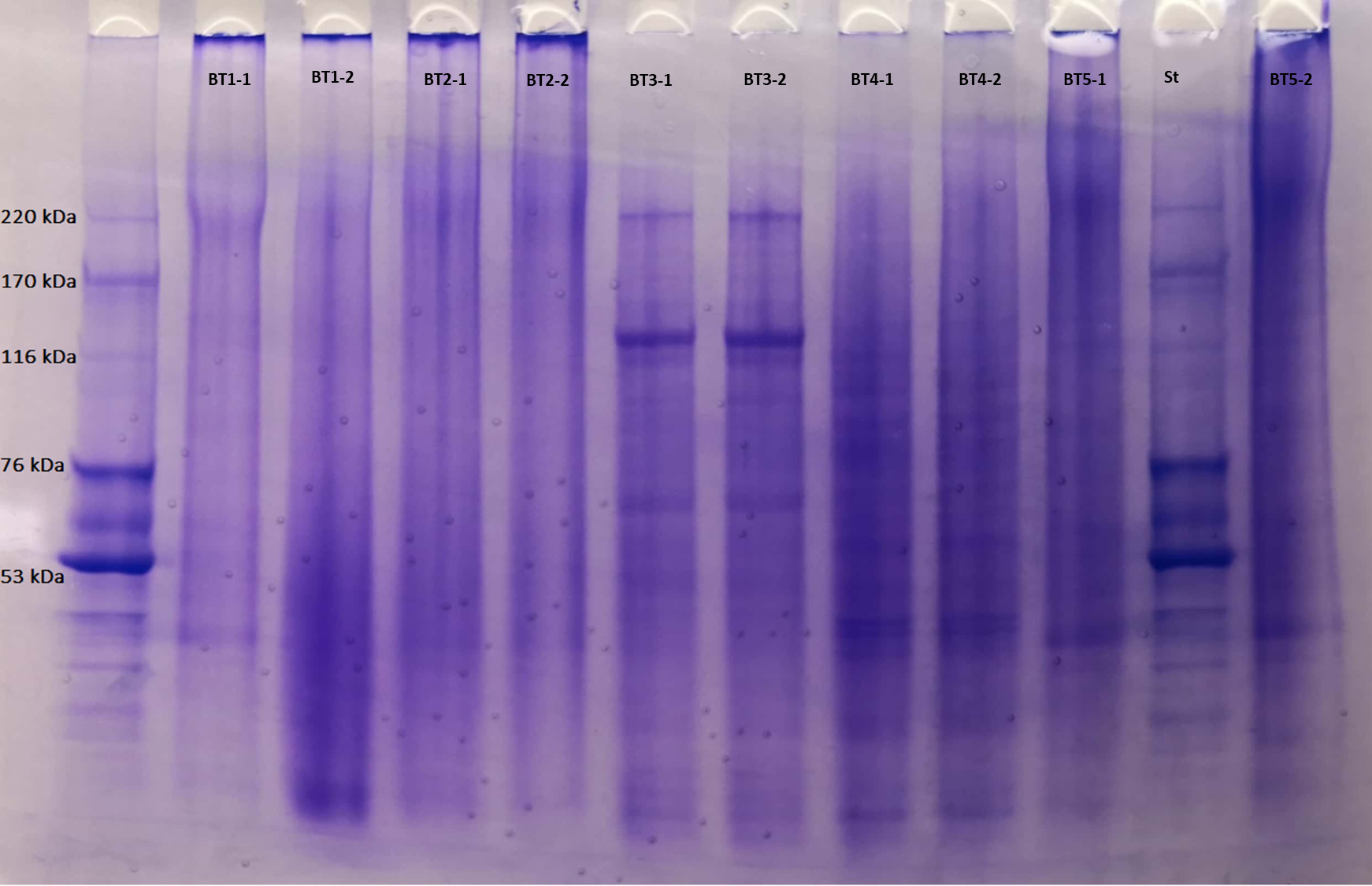


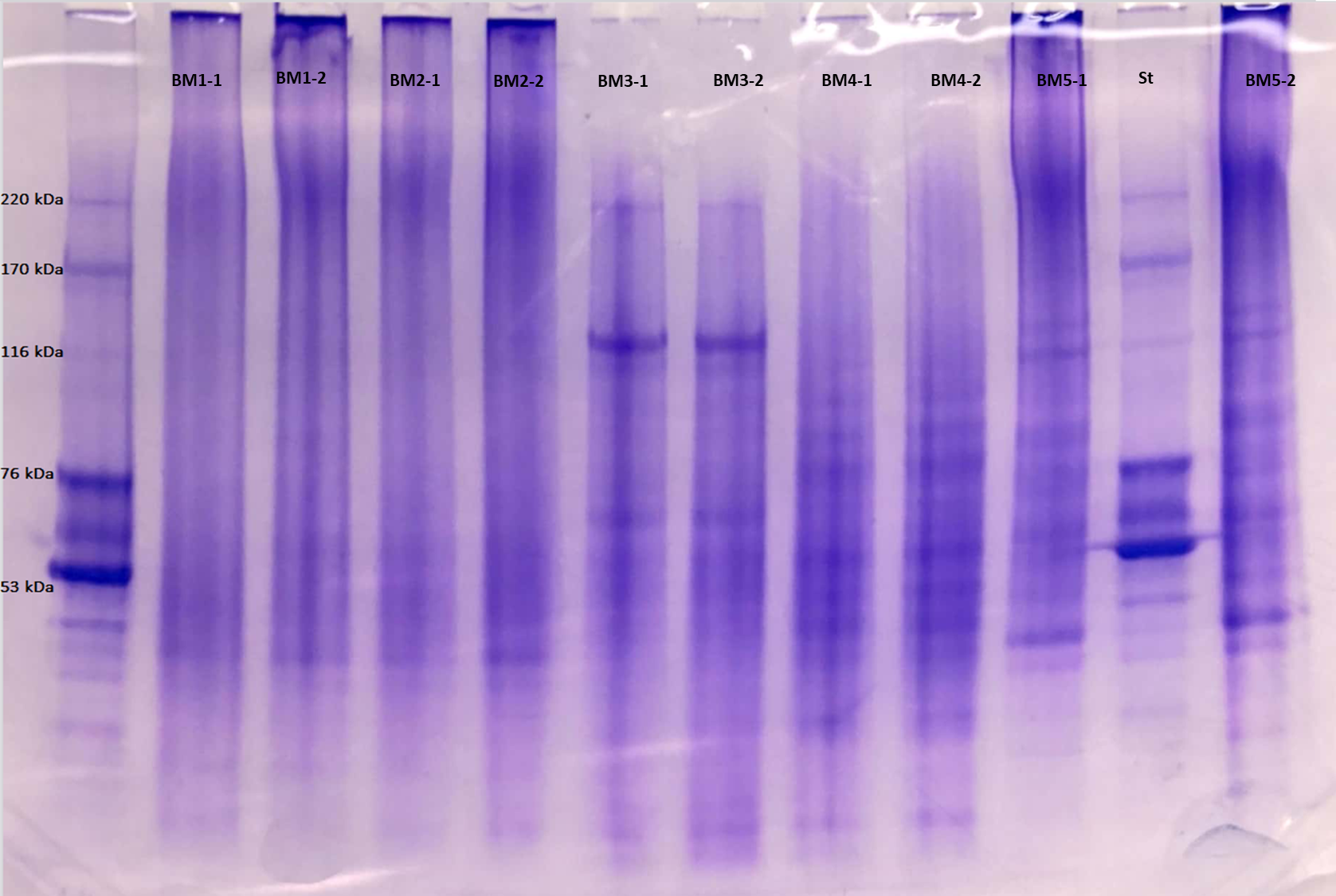

Supplement: Multimedia component 1 [file mmc1.docx]
